# Evaluation of the fibroblast growth factor system as a potential target for therapy in human prostate cancer

**DOI:** 10.1038/sj.bjc.6602274

**Published:** 2005-01-18

**Authors:** B Gowardhan, D A Douglas, M E Mathers, A B McKie, S R C McCracken, C N Robson, H Y Leung

**Affiliations:** 1 Urology Research Group, Northern Institute for Cancer Research, University of Newcastle, Newcastle upon Tyne, NE2 4HH, UK

**Keywords:** prostate cancer, FGFR3, FGFR4, sFGFR, paclitaxel, *γ*-irradiation

## Abstract

Overexpression of fibroblast growth factors (FGFs) has been implicated in prostate carcinogenesis. FGFs function via their high-affinity interactions with receptor tyrosine kinases, FGFR1–4. Expression of FGFR1 and FGFR2 in prostate cancer (CaP) was not found to be associated with clinical parameters. In this report, we further investigated for abnormal FGFR expression in prostate cancer and explore their significance as a potential target for therapy. The expression levels of FGFR3 and FGFR4 in CaP were examined and corroborated to clinical parameters. FGFR3 immunoreactivity in benign prostatic hyperplasia (BPH) and CaP (*n*=26 and 57, respectively) had similar intensity and pattern. Overall, FGFR4 expression was significantly upregulated in CaP when compared to BPH. A significant positive correlation between FGFR4 expression and Gleason score was noted: Gleason score 7–10 tumours compared to BPH (*P*<0.0001, Fisher's exact test), Gleason score 4–6 tumours compared to BPH (*P*<0.0004), and Gleason 7–10 compared to Gleason 4–6 tumours (*P*<0.005). FGFR4 overexpression was associated with an unfavourable outcome with decreased disease-specific survival (*P*<0.04, log rank test). FGF-induced signalling is targeted using soluble FGF receptor (sFGFR), potent inhibitor of FGFR function. We have previously shown that sFGFR expression via a replication-deficient adenoviral vector (AdlllcRl) suppresses *in vitro* FGF-induced signalling and function in human CaP DU145 cells. We tested the significance of inhibiting FGF function along with conventional therapeutic modalities in CaP, and confirmed synergistic effects on *in vitro* cell growth (proliferation and colony formation) by combining sFGFR expression and treatment with either Paclitaxel (Taxol®) or *γ*-irradiation. In summary, our data support the model of FGF system as valid target for therapy in CaP.

Prostate cancer is the commonest cancer in men and the second commonest cause of cancer-related death in men, and its incidence is increasing (Woolf, 1995; Boyle *et al*, 1996). Prostate cancer is an enigmatic disease. It is histologically present in 80% of men over the age of 80 years, but will only clinically manifest itself in about 10%. Increasing use of serum measurement of prostate-specific antigen is facilitating early diagnosis of prostate cancer. There are currently limited prognostic markers that may allow patients found to have early prostate cancer to be stratified into different management plans. Hence, new methods of predicting disease progression are urgently needed.

Abnormal expression of peptide growth factors and their high-affinity receptor tyrosine kinases are important in the development and progression of prostate cancer. These mitogens enhance tumour proliferation and invasion while inhibiting apoptosis. Several peptide growth factors have been implicated in prostate cancer development and progression, including insulin-like growth factors, epidermal growth factor and members of the fibroblast growth factors (Byrne *et al*, 1996; Tennant *et al*, 1996; Dorkin *et al*, 1999a).

The family of fibroblast growth factors (FGFs) and their receptors (FGFRs) are important in the prostate organogenesis as well as the pathogenesis of prostate cancer (Cunha *et al*, 1987; Leung *et al*, 1996; Ittmann and Mansukhani, 1997; Dorkin *et al*, 1999b). Fibroblast growth factors make up a large family of 23 related polypeptides with highly conserved amino-acid sequences, sharing 13–71% sequence homology, ranging from 17 to 34 kDa in molecular weight (Ornitz and Itoh, 2001; Yamashita *et al*, 2000). The FGF family interacts directly with heparin and heparin-like glycosaminoglycans. This provides essential functions in stabilising FGFs and facilitating effective interaction between FGF and FGFR (Mansukhani *et al*, 1992; Roghani *et al*, 1994).

FGFRs are transmembrane receptor tyrosine kinases. Upon ligand binding, FGFRs undergo dimerisation and transphosphorylation at the intracellular kinase domain. Four FGFR genes have been cloned in humans and they share a 55–72% sequence homology (Jaye *et al*, 1992; Powers *et al*, 2000). FGFR proteins are characterised by three immunoglobulin (Ig)-like domains (designated loops I, II, and III) within the extracellular region, a single transmembrane region and a split cytoplasmic tyrosine kinase domain. Only loops II and III are required for FGF binding, while the C-terminal portion of loop III determines the ligand specificity. Different receptor isoforms arise due to alternate splicing of exons coding for Ig loop III (Ornitz *et al*, 1996). For example, alternative splicing of FGFRl results in isoforms designated FGFRlIIIb and FGFRlIIIc, which have differential FGF binding characteristics. FGFRlIIIb binds efficiently to aFGF, FGF3 and FGF 10, while FGFRl Ilk binds to aFGF, bFGF, FGF4, FGF6, FGF8 and FGF9 (Ornitz *et al*, 1996). FGFRl-3 demonstrate both IIIb and IIIc splice variant isoforms, but FGFR4 is unique and has no IIIb splice variant, being expressed as the IIIc isoform only (Johnson *et al*, 1991; Vainikka *et al*, 1992; Chellaiah *et al*, 1994).

Soluble FGFR, containing the extracellular ligand-binding domain of the native FGFR, is secreted and without a tyrosine kinase domain. A number of studies have assessed the inhibitory effect of the tyrosine kinase-deficient growth factor receptors (FGFR and vascular endothelial growth factor receptor (VEGFR) (Celli *et al*, 1998; Ozen *et al*, 2001; Mahasreshti *et al*, 2001; Ogawa *et al*, 2002; Becker *et al*, 2002). The main characteristics of these soluble growth factor receptors include the ability to bind ligand in the extracellular space, thus ‘mopping up’ the ligands preventing them from binding to the native full-length receptors, and to dimerise with the native receptors forming inactive homo- or hetero- dimers, thus blocking ligand-induced cellular signalling and function. Celli *et al*, using a soluble FGFR 2 construct, demonstrated inhibition of FGF-induced signalling, resulting in lethal defects in organogenesis. The soluble form of the FGFR was found to be more potent than the membrane-bound form (Celli *et al*, 1998). Using adenoviral-mediated soluble FGFRlIIIc expression, work from our laboratory have previously demonstrated significant suppression of FGF-induced signalling and function (proliferation and invasion) in human prostate cancer DU145 cells (Gowardhan *et al*, 2004). The efficacy of such an approach remains to be tested against chemotherapy and radiotherapy, modalities of cancer treatment in current clinical practice.

FGFR1 is overexpressed in prostate cancer, and FGFR2 has been detected in both prostate cancer and benign prostatic hyperplasia (Leung *et al*, 1997; Giri *et al*, 1999). However, neither FGFR1 nor FGFR2 expression in prostate cancer was noted to have any significant correlation to clinical parameters including tumour grade, stage, and outcome on disease survival. The objectives of this study were two-fold. First, we examined for abnormal expression of FGFR3 and FGFR4 in clinical prostate cancer specimens. Second, the novel approach of targeting the FGF system in combination with chemotherapy (paclitaxel) or radiation therapy (*γ*-irradiation) was tested using an *in vitro* DU145 prostate cancer cell model.

## MATERIALS AND METHODS

### Patients and samples

Archival prostate specimens from cases of newly diagnosed prostate cancer were obtained from transurethral resection of the prostate gland (TURP). The specimens were formalin fixed and paraffin embedded. Sections were prepared and mounted on APES-coated slides. A total of 57 cases of prostate cancer and 26 cases of benign prostate hyperplasia were selected. The age range of the cancer group was 56–86 years (mean age 71 years) at the time of diagnosis. Bone scans were performed in 50 of these 57 patients. In total, 21 of the bone scans were positive, signifying the presence of bony metastases. A summary of the relevant demographic data is presented in Table 1.

### Immunohistochemistry

Fibroblast growth factor receptors 3 and 4 protein expression levels were examined using immunohistochemistry. Prior to commencing staining of the prostate specimens, the use of FGFR3- and FGFR4-specific antibodies were optimised on serial prostate sections to demonstrate clean and reproducible signals. The antibodies were also tested by Western blotting to show specific band of the correct molecular weight for each receptor (data not shown). The slides were dewaxed in xylene prior to rehydration in 100, 70 and 50% ethanol and finally water. Endogenous peroxidase activity was blocked with 30% hydrogen peroxide diluted 1 : 60 in methanol, then placed in water prior to antigen retrieval using pressure cooking in a 0.01 M citrate buffer (pH=6.0) for 6 min. The slides were then placed in phosphate-buffered saline (PBS), before blocking with 10% swine serum in PBS for 20 min. The primary antibodies were then applied at 1 : 200 (5 *μ*g ml^−1^) for FGFR3 and 1 : 200 (5 *μ*g ml^−1^) for FGFR4 and incubated at 4°C overnight, (rabbit anti-FGFR3 and FGFR4 polyclonal IgG; Santa Cruz, USA). Sections were also incubated in PBS alone as a negative control. The slides were then washed in PBS before incubation with the secondary biotinylated swine anti-rabbit IgG antibody (Dako, UK) diluted at 1 : 250 in PBS for 30 min at room temperature. Following further washings in PBS, visualisation of immunoreactivity was performed using Vectastain Avidin Biotin Complex Kit (Vector Laboratories, UK), according to the manufacturer's instructions. Finally, the slides were treated with DAB (3′3′-diaminobenzidetetrahydrochloride), and then counterstained with Harris haematoxylin, before dehydration with graded ethanol and xylene prior to being mounted with cover slide.

### Scoring of slide sections

The slides were viewed by light microscopy and scored for staining intensity of FGFR3 and FGFR4. Two independent observers (DAD and MEM) scored all sections, with no knowledge of the clinical parameters for each section at the time of scoring. The scoring was semiquantitative looking for presence and intensity of staining. FGFR3 and FGFR4 immunoreactivity was considered positive if more than 25% of the section was stained. Intensity of the staining was graded as negative (0), weak (1), moderate (2), and strong (3) as previously published (Armes *et al*, 1999; Bouras *et al*, 2001).

### Cells, cell culture, and treatment with paclitaxel or *γ*-irradiation

Cultured cells were maintained in growth medium (RPMI 1640 (Gibco BRL, Invirrogen, Paisley, UK), containing HEPES buffer (25 mM) and L-glutamine (20 mM)), supplemented with 10% heat-inactivated fetal calf serum (Sigma-Aldrich, Dorset, UK), 100 U ml^−1^ of penicillin, and 100 *μ*g ml^−1^ of streptomycin (Gibco BRL); this was referred to as full medium. The androgen-unresponsive human prostate carcinoma DU145 cell line was purchased from the American Type Culture Collection (ATCC, Rockville, MD, USA). The 293T cell line (El-transformed human embryonic kidney cells) was a kind gift from Professor A Sharrocks (Manchester University, UK) and used as a packaging cell line for adenoviruses. Paclitaxel (Taxol®) (Sigma-Aldrich, Dorset, UK) was reconstituted to a final concentration of l mmol ml^−1^. *γ*-Irradiation was delivered at a dose of 3.26 Gy min^−1^.

### Preparation of the soluble FGFR1 gene construct

The details of the preparation of the soluble FGFR1 gene construct have been previously described (Li *et al*, 2002). Briefly, the soluble FGFR1 gene was cloned using polymerase chain reaction (PCR) and incorporated into the adenoviral AdTrack vector, which is a shuttle vector containing a GFP expression cassette and two cytomegaloviral promoter regions. The recombinant AdTrack vector was cotransformed with the adenoviral backbone vector AdEasy to yield the recombinant adenoviral construct (AdIIIcRl). The construct was amplified by successive transfections/infections in HEK293T cells and the viral particles harvested by five cycles of freeze–thawing. The viruses were purified using CsCl and titrated using the tissue culture infectious dose 50 (TCID50) method. Similarly, control empty adenoviruses without the soluble FGFR1 gene were constructed (AdE).

### Proliferation assay

A total of 3000 DU145 cells per well were seeded out in 96-well plates and allowed to grow for 24 h in full medium. Typically, the cells were 70% confluent at 24 h, and the medium was replaced with a 10 *μ*l of full medium with or without adenovirus stock (AdIIIcRl or AdE; 100 viral particles per cell (p.p.c.) respectively). Plates were then incubated for 3 h at 37°C, 5% CO2 with gentle shaking to allow maximum contact of virus with all cells. The cells were maintained in full medium with Paclitaxel at doses of 0, 2.5, 5, 7.5 and 10 nmol ml^−1^, for 5 days at 37°C, 5% CO_2_. At this point, 10 *μ*l of (4-[3-(4-iodophenyl)-2-(4-nitrophenyl)-2H-5-tetrazolio]-1,3-benzene disulphonate) (WST-1, Roche, Welwyn Garden City, Hertfordshire, UK) was added to each well and incubated at 37°C, 5% CO2 for 3 h. The plate were read using an ELISA reader at a wavelength of 450 nm. As a further control, we used cells treated with the same doses of paclitaxel but without adenoviruses.

In a separate experiment, 3 × 10^4^ DU145 cells were resuspended in 1 ml of full media per sterile universal container. They were then exposed to *γ*-irradiation at doses of 0, 2,4, 6, 8 and 10 Gy. The cells were then seeded out in a 96-well plate at a concentration of 3000 cells per well in 100 *μ*l of full medium. After 24 h, the medium was removed and was replaced with a 10 *μ*l of full medium with or without adenovirus stock (AdIIIcRl or AdE; 100 viral p.p.c., respectively). Plates were then incubated for 3 h at 37°C, 5% CO_2_ after which 90 *μ*l of full media were added to make up a final volume of 100 *μ*l. The plates were then incubated for 5 days. At this point, 10 *μ*l of (4-[3-(4-iodophenyl)-2-(4-nitrophenyl)-2H-5-tetrazolio]-1,3-benzene disulphonate) (WST-1, Roche) was added to each well and incubated at 37°C, 5% CO_2_ for 3 h. The plate was read using an ELISA reader at a wavelength of 450 nm. As a further control, we used cells treated with the same doses of *γ*-irradiation but without adenoviruses. Both experiments were performed in quadruplet and repeated three times.

### Colony-forming assay

In total, 5000 DU145 cells were seeded out in T25 flasks in 6 ml of full media. After 24 h, the medium was removed and adenoviral stock at a dose of 100 viral p.p.c. (AdlllcRl or AdE) in 1 ml of full media was added and the flasks incubated for 3 h at 37°C, 5% CO_2_ on a shaker. Amounts of 5 ml of full medium containing paclitaxel at doses of 0, 1, 2.5, 5, 7.5 and 10 was then added to make up a final volume of 6 ml. The plates were then incubated for 14 days at 37°C, 5% CO2 without changing the media. As a further control, cells treated with paclitaxel but without adenoviruses were used. After 14 days, the medium was removed; cells washed gently with sterile PBS, and fixed with methanol : acetic acid (3 : 1) for 30 min at room temperature. The fixing reagent was removed and 0.4% Methylene blue added to stain the colonies for 30 min. The methylene blue was removed and colonies washed gently with water to remove excess stain. The colonies were then counted. The experiment was repeated three times.

In a separate experiment, 5000 DU145 cells were resuspended in 1 ml of full medium in sterile universal containers. They were then exposed to 0, 2. 4, 6, 8 and 10 Gy of *γ*-irradiation. The cells were then seeded out in T25 flasks and incubated for 24 h at 37°C, 5% CO_2_. After 24 h, the medium was removed and replaced with adenoviral stock (AdlllcRl or AdE) at a dose of 100 viral p.p.c. in 1 ml of full medium. The plates were incubated for 3 h at 37°C, 5% CO_2_ with gentle shaking. Amounts of 5 ml of full medium was then added to make up a final volume of 6 ml. The plates were then incubated for 14 days at 37°C, 5% CO_2_ without changing the medium. As a further control, cells exposed to *γ*-irradiation but without adenoviruses were used. After 14 days, the medium was removed; cells washed gently with sterile PBS, and the cells fixed with methanol : acetic acid (3 : 1) for 30 min at room temperature. The fixing reagent was removed and 0.4% methylene blue added to stain the colonies for 30 min. The methylene blue was removed and colonies washed gently with water to remove excess stain. The colonies were then counted. The experiment was repeated three times.

### Statistical analysis

Immunoreactivity of FGFR3 and FGFR4 in prostate carcinoma and BPH was analysed using Fisher's exact test of probability. To analyse patient survival compared to FGFR expression, Kaplan–Meier survival curves were plotted and the difference in survival between different groups assessed using the log rank test. To perform these tests the statistical package Arcus Quickstat (Biomedical Version 1.1) was used.

Differences in mean values of absorbance on WST-1 assay and number of colonies in the colony-forming assay were evaluated using the Student's *t*-test for unpaired data. A probability value of less than 0.05 was taken to indicate statistical significance.

## RESULTS

### FGFR3 expression is not upregulated in prostate cancer

Immunoreactivity for FGFR3 was observed in majority (>95%) of the cases examined, both specimens from prostate carcinoma and BPH. The staining pattern was uniform throughout the epithelium with moderate to strong staining intensity. In contrast, the stroma expressed FGFR3 at low levels. Epithelial staining was observed to be both cytoplasmic and nuclear, with moderate immunoreactivity in the cytoplasm and strong signals in the nucleus.

**Figure 1:**
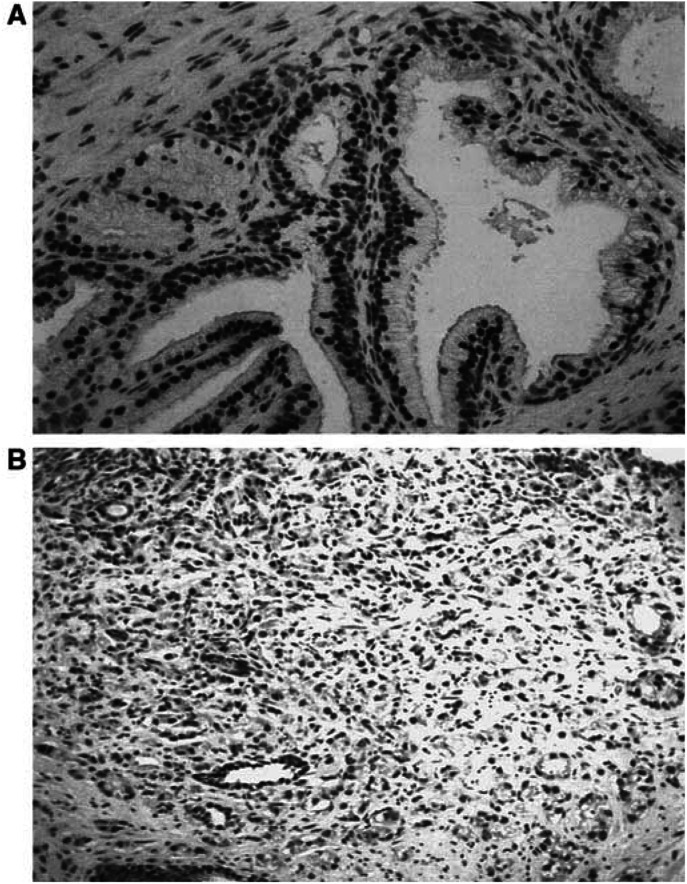
Moderate levels of FGFR3 expression are present in both BPH and prostate cancer: (**A**) BPH, (**B**) a case of high-grade prostate cancer. FGFR3 immunoreactivity is predominantly epithelial, with only weak stromal signals. FGFR3 expression in the prostate epithelium exhibits a combination of nuclear and cytoplasmic localisation.

The intensity of immunoreactivity was compared between the BPH and prostate carcinoma specimens. No difference in overall expression of FGFR3 was found between benign and malignant prostate epithelium (Figure 1). The relative cytoplasmic and nuclear FGFR3 signals in benign and malignant prostate epithelium were similar.

### FGFR4 expression in human prostate cancer

**Figure 2:**
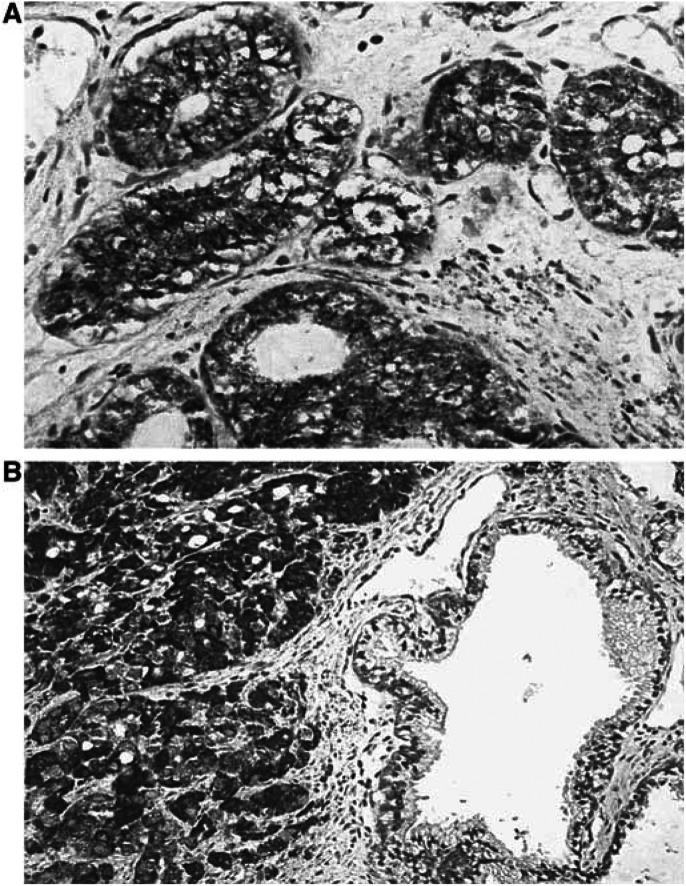
(**A**) FGFR4 immunoreactivity in a Gleason 8 prostate cancer specimen, showing moderate to strong FGFR4 immunoreactivity. The staining is predominantly epithelial and within the epithelium, there is no nuclear expression of FGFR4. There appears to be increased staining at the cell membrane, in keeping with the transmembranous nature of the FGFR4. (**B**) Comparison of FGFR4 staining in a benign gland next to an area of prostate cancer (Gleason 9), on the same tissue section. This figure shows increased FGFR4 expression in the malignant epithelium, in contrast to the adjacent benign prostate epithelium with no detectable signals.

FGFR4 immunoreactivity was seen in both benign and malignant prostate sections. Signals observed in the stroma were scanty and at low intensity. The malignant epithelium showed uniform moderate to strong immunoreactivity for FGFR4. FGFR4 staining was entirely cytoplasmic, with no nuclear signals. In some sections, there was also convincing membranous staining in keeping with a transmembranous localisation of FGFR4 (Figure 2).

**Figure 3:**
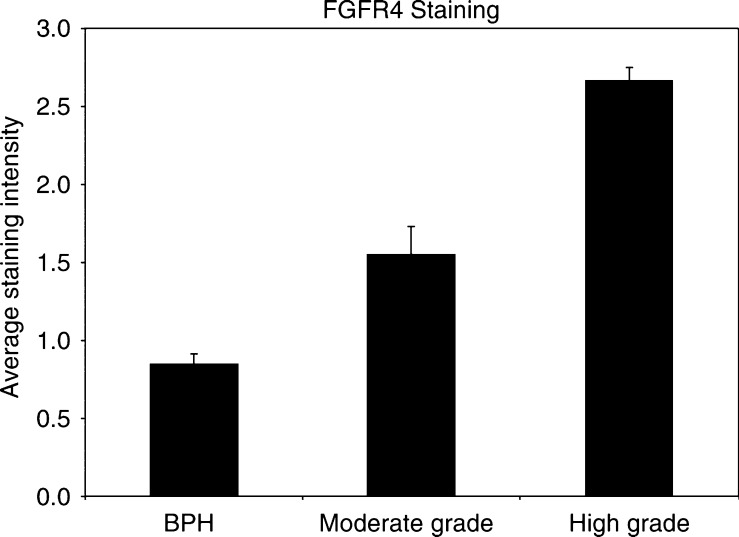
Graph showing the different staining intensities for FGFR4, exhibited between benign prostate and moderate/high-grade prostate cancer. Moderate-grade cancer had significantly higher expression of FGFR4 compared to BPH (*P*<0.0004), as did high-grade cancer (*P*<0.00001). Increased expression of FGFR4 was also shown when comparing moderate to high-grade cancers (*P*<0.005).

FGFR4 immunoreactivity was compared between BPH and various grades of prostate cancer. The staining intensity was increased in cancer compared to BPH. The prostate cancer specimens were divided into low to moderate- and high-grade disease, representing Gleason sum scores of <7 and 7–10, respectively. The scores for each group were analysed using Fisher's exact test. Both low to moderate-grade and high-grade tumours had significantly higher expression of FGFR4 than BPH (*P*<0.0004 and <0.0000l, respectively). High-grade prostate cancer also had significantly higher expression of FGFR4 protein than moderate-grade prostate cancer (*P*<0.005) (Figure 3).

**Figure 4:**
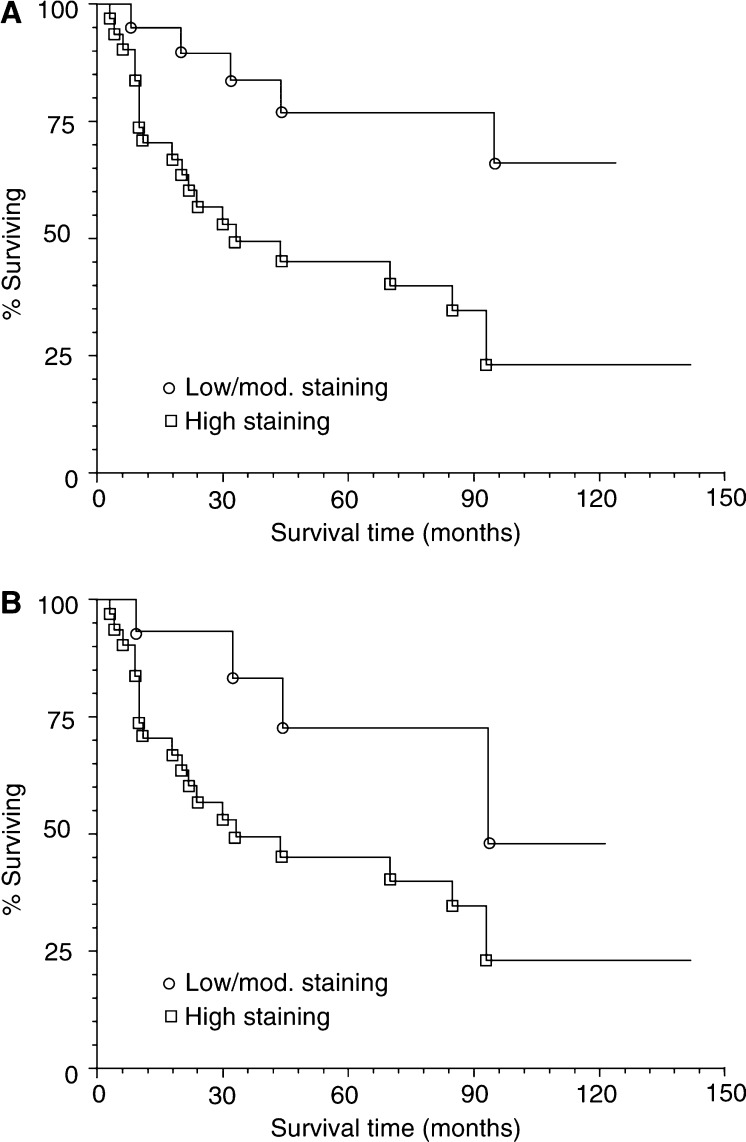
FGFR4 expression compared to patient survival. (**A**) Kaplan–Meier plot comparing survival in patients with high levels of FGFR4 expression compared to patients with low to moderate staining for FGFR4. Data from all patients with prostate cancer, moderate and high-grade cancer, was included in this plot. Patients with high expression of FGFR4 had a decreased disease-specific survival time than patients with low to moderate FGFR4 expression (*P*<0.0006. log rank test). (**B**) Kaplan–Meier plot comparing survival in patients with high Gleason sum score tumours. Patients with high expression of FGFR4 in this group also had a decreased survival time compared to low to moderate FGFR4 expression (*P*<0.036, log rank test).

Increased FGFR4 immunoreactivity was significantly associated with decreased patient survival. In the cancer group, 51 patients had informative survival data. FGFR4 overexpression was associated with less favourable disease-specific survival (*P*<0.006, Figure 4A). In this group, patients with low to moderate staining for FGFR4 had a mean survival time of 64.6 months, compared to 45.5 months for patients with prostate cancer expressing high levels of FGFR4. Among patients with high-grade disease (Gleason score 8–10), high levels of FGFR4 expression was weakly associated with decreased survival time (*P*<0.04, Figure 4B). Patients with high-grade prostate cancer and low to moderate staining had a mean survival of 54.4 months compared to 45.5 months for people with high FGFR4 expression in high-grade prostate cancer. FGFR4 expression was not noted to be associated with tumour stage, serum PSA or the presence of bony metastases.

### Synergistic effects of combined treatment on *in vitro* proliferation

**Figure 5:**
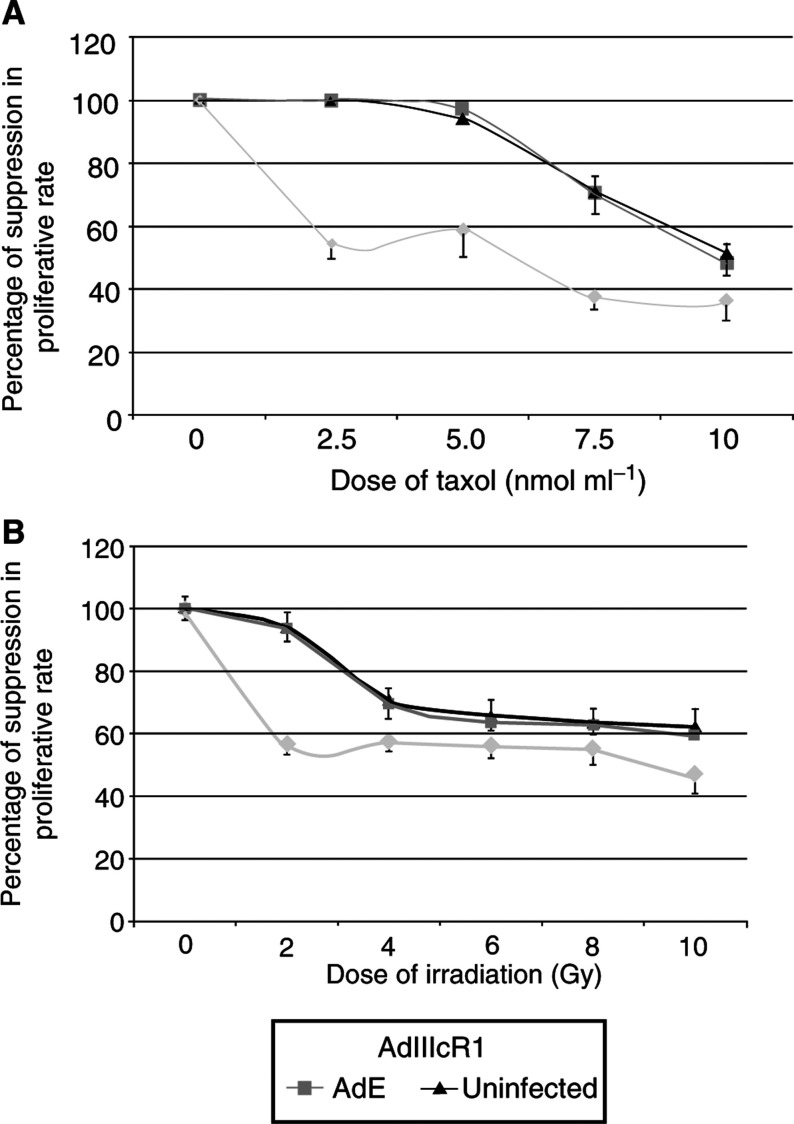
Combination treatment on proliferation of DU145 cells. (**A**) *soluble FGFR*+*paclitaxel*: Graph showing a suppression of proliferation by 1% by paclitaxel when used alone at a dose of 2.5 nmol ml^−1^ as compared to a suppression of 45% when used in combination with sFGFR (*P*=0.005). The synergism of the combined treatment is present throughout the dose range (AdIIIcRl=recombinant adenovirus with soluble FGFRl gene; AdE=empty adenovirus). Even at a dose of 10 nmol ml^−1^ the suppression by the combination is significant (*P*=0.049). (**B**) *soluble FGFR*+γ-*irradiation*: Graph showing a suppression of proliferation by 8% by *γ*-irradiation when used alone at a dose of 2 Gy as compared to 45% when used in combination with soluble FGFR (*P*=0.0003). The synergism of the combined treatment is present throughout the dose range (AdIIIcRl=adenovirus with soluble FGFRl gene; AdE=empty adenovirus). At a dose of 10 Gy the suppression continues to be more significant than single therapy (*P*=0.004).

AdIIIcRl used on its own caused a suppression of proliferation by 30% in full medium compared to untreated controls. Paclitaxel when used alone caused a suppression of only 1% at a dose of 2.5 nmol ml^−1^ compared to untreated controls. When the two were combined the suppression in proliferation was 45% (*P*=0.005) compared to untreated controls. This synergism was noted throughout the dose range of paclitaxel with a suppression of 63% at 10 nmol ml^−1^ compared to 49% for paclitaxel alone at 10 nmol ml^−1^ (*P*=0.049) (Figure 5A). Overall, IC50 for Paclitaxel was reduced from 10 nmol ml^−1^ (alone) to 2.5 nmol ml^−1^ (combined with soluble FGFR expression).

Similarly, for *γ*-irradiation alone treated cells, the suppression in proliferation was 8% at a dose of 2 Gy compared to untreated cells. The combination of AdIIIcRl and *γ*-irradiation brought about a suppression of 45% compared to untreated cells (*P*=0.0003). As with paclitaxel, this suggested a synergism that existed throughout the dose range. At a higher dose of 10 Gy, the combined treatment suppressed proliferation by 55% as compared to 39% for 10 Gy of *γ*-irradiation alone (*P*=0.004) (Figure 5B). With *γ*-irradiation the IC50 was reduced from 10 Gy (single agent) to 8 Gy (combined).

### Synergistic effects of combined treatment on *in vitro* colony formation

**Figure 6:**
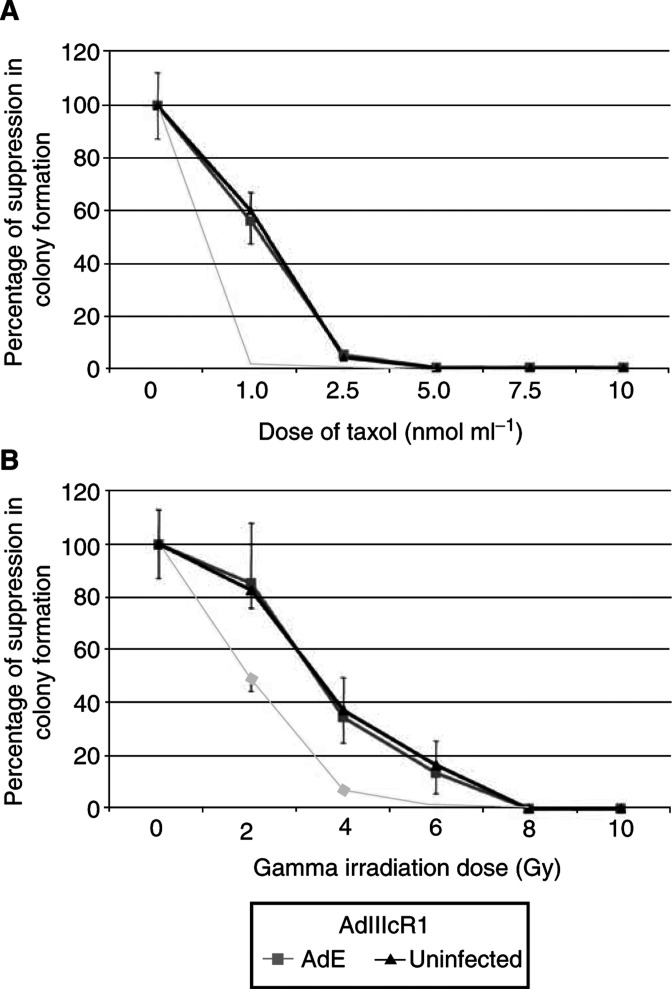
Combination treatment on colony formation of DU145 cells. (**A**) *soluble FGFR*+*paclitaxel*: Graph showing a suppression of proliferation by 40% by paclitaxel when used alone at a dose of 1 nmol ml^−1^ as compared to 99% when combined with soluble FGFR (*P*=0.0007). There was a complete suppression in colony formation with a dose of 2.5 nmol ml^−1^ when the two treatments were used in combination (AdIIIcRl=recombinant adenovirus with soluble FGFRl gene; AdE=empty adenovirus). (**B**) *soluble FGFR*+γ-*irradiation*: Graph showing a suppression of colony formation by 13% when *γ*-irradiation is used alone as compared to 50% when combined with soluble FGFR (*P*=0.0001). There was a near-complete suppression in colony formation with the combined treatment with a dose of 6 Gy of *γ*-irradiation (*P*=0.003) (AdIIIcRl=recombinant adenovirus with soluble FGFRl gene; AdE=empty adenovirus).

AdIIIcRl when used alone caused a suppression of colony formation by 43% compared to untreated controls. Paclitaxel used alone at a paclitaxel dose of 1 nmol ml^−1^ caused suppression in colony formation by 40% as compared to untreated controls. When the two treatments were combined, the suppression was 99% at a dose of 1 nmol ml^−1^ (*P*=0.0007). With paclitaxel alone, the colony formation was completely suppressed by a dose of 5 nmol ml^−1^, whereas with the combination complete suppression was noted at a dose of 2.5 nmol ml^−1^) (Figure 6A). Furthermore, the IC50 of paclitaxel for colony formation was reduced from 1.25 to 0.5 nmol ml^−1^. This suggested a potent synergism between the two treatment modalities.

When *γ*-irradiation was used alone, the suppression in colony formation was 13% as compared to untreated controls. When used in combination with AdIIIcR1, the suppression was 50% as compared to untreated controls (*P*=0.0001). This synergistic effect continued with the increasing dose of *γ*-irradiation. At a dose of 6 Gy, the combined treatment resulted in suppression of colony formation by 99% compared to untreated controls while *γ*-irradiation used alone at 6 Gy caused a suppression by 85% (*P*=0.003). By a dose of 8 Gy, both *γ*-irradiation used alone and the combination treatment caused a complete suppression in colony formation. With *γ*-irradiation the IC50 for colony formation was reduced from 5 to 2 Gy) (Figure 6B).

## DISCUSSION

Overexpression of multiple FGFs (namely aFGF/FGFl, bFGF/FGF2, FGF6 and FGF8) has been identified in prostate cancer (Leung *et al*, 1996; Ittmann and Mansukhani, 1997; Dorkin *et al*, 1999b; Ropiquet *et al*, 2000). FGF8 appeared to be particularly important as paracrine and autocrine factors in prostate cancer (Leung *et al*, 1996, 1997). FGF8 expression was significantly associated with tumour grade and stage, and was a predictor of disease-specific survival in patients followed up for over 10 years (Dorkin *et al*, 1999a). Of the four FGFRs, expression of FGFR1 and FGFR2 has been examined in resected prostate cancer specimens. They both appeared to be expressed in prostate cancer; however, a significant correlation between their expression and clinicopathologic parameters has not been observed.

In this study, we have examined the levels of expression of FGFR3 and FGFR4 proteins in resected prostate specimens. We showed that FGFR3 is expressed in the majority of BPH and prostate cancer. The expression pattern was mainly epithelial with predominant nuclear signals in both BPH and malignant prostate. The FGFR3 is a transmembranous receptor but nuclear expression has previously been described. Several FGFs and FGFR1 and FGFR3 have been shown to be present within the cell nucleus (Feng *et al*, 1996; Kilkenny and Hill, 1996; Stachowiak *et al*, 1997). The presence of FGF1 in the nucleus is necessary for maximal mitogenic response (Mehta *et al*, 1998). FGFR1 has been shown to adopt a perinuclear location on ligand activation and also associates with nuclear matrix and nucleoplasm upon FGF2 induction (Prudovsky *et al*, 1994; Maher, 1996; Stachowiak *et al*, 1996).

FGFR3 binds to multiple FGFs known to be upregulated in human prostate cancer (FGF1, FGF2 and FGF8), and is potentially important in prostate cancer. However, we did not observe any significant change in the levels of FGFR3 expression between BPH and prostate cancer. A more subtle shift in FGFR3 expression from the cytoplasm to the nucleus, which has been observed in breast cancer, is not present in prostate cancer (Zammit *et al*, 2001). FGFR3 has previously been shown to be the dominant FGFR in prostate epithelium (Ittmann and Mansukhani, 1997). Hence, we conclude that there is no significant change in the overall expression and localisation of the FGFR3 in human prostate cancer. It would therefore seem unlikely that FGFR3 plays a key role in prostate carcinogenesis.

Abnormal expression of members of the family of FGF and receptor represents an appealing target for therapy. Besides FGFR expression analysis, we describe synergistic effects of combining soluble FGFR gene therapy with either paclitaxel or *γ*-irradiation on *in vitro* growth of DU145 prostate cancer cells. Proliferation was suppressed by 30, 1 and 8%, respectively, by soluble FGFR (at 100 viral particles per cell (data not shown)). paclitaxel (at 2.5 nmol ml^−1^) and *γ*-irradiation (at 2 Gy) when used alone. Proliferation was suppressed by 45% with the combined treatment of soluble FGFR+paclitaxel or sFGFR+*γ*-irradiation (*P*=0.005 and 0.0003, respectively) with paclitaxel and *γ*-irradiation used at the lowest dose. We further evaluated the synergism in a colony-forming assay using DU145 cells. With single treatment with soluble FGFR (100 viral particles per cell (data not shown)), paclitaxel (1 nmol ml^−1^) and *γ*-irradiation (2 Gy), suppression in colony formation was 43, 40 and 13%, respectively. When the treatments were combined, the suppression was 99 and 50%, respectively (*P*=0.0007 and 0.0001 respectively) at 1 nmol ml^−1^ of paclitaxel and 2 Gy of *γ*-irradiation. The combined treatment caused a near-complete suppression of colony formation at a lower dose than the individual treatments. Hence, the suppression of FGFR function along with either chemotherapy or radiation therapy may potentially reduce the dose of chemotherapy or radiotherapy required to achieve therapeutic success.

Adenoviral-mediated transgene delivery has been combined with both chemotherapy and radiotherapy in a number of studies and have been shown to be synergistic, either as a sensitizing agent or indeed as a means of enhancing transgene expression, both of which are useful in the treatment of cancers (Zeng *et al*, 1997; Seidman *et al*, 2001; Li *et al*, 2002). The combination of the two agents is thought to be beneficial in a number of ways, one of which is to reduce the dose of either the chemotherapeutic agent or of radiotherapy. This will, undoubtedly, be of benefit in reducing toxicity to patients. It is also known that ionising radiation improves transfection/transduction efficiency and transgene integration (Zeng *et al*, 1997). Gene therapy and radiation therapy target best at different parts of the cell cycle, that is, gene therapy requires the ‘S’ phase of the cell cycle while ‘M’ and ‘G_2_’ phases are most radiosensitive (Simons and Marshall, 1998). Phosphorylated prodrugs such as ganciclovir, acyclovir or valacyclovir are incorporated into the newly synthesised DNA causing termination of DNA synthesis and, thus, cell death. This may increase the DNA susceptibility to radiation damage. Also, by incorporation into the DNA, phosphorylated prodrugs may interfere with repair of radiation-induced DNA damage. It is thought that radiation may also enhance the ‘bystander effect’ of gene therapy. This maybe due to the release of products from the radiation-damaged cells and the efficient uptake and presentation of tumour antigens by immune effector cells attracting immunocytes and mediating an antitumour response (Teh *et al*, 2002).

In summary, we presented evidence for clinical significant FGFR4 overexpression in prostate cancer, and further validated the potential of targeting the FGFR system for treatment in conjunction with current available modalities.

## Figures and Tables

**Table 1:** Clinical details

	**Total number (*n*=38)** a	**Metastases at diagnosis** b	**Mean serum PSA**	**PSA range**	**No. with PSA data (*n*=21)** c
*Stage*					
Tl	12	1/12	17.1	2.7–45	5
T2	11	6/11	271.2	3.6–1436	6
T3	9	4/6	333.6	5.7–2000	7
T4	6	5/6	79.0	3.6–136	3

*Gleason score*	(*n*=57)				
2–6	9	1/9	67.2	2.7–154	3
7	7	1/6	16.7	3.6–26.9	4
8–10	41	19/35	286.9	3.6–2000	14

a T stage at diagnosis was not available for 19 of the prostate carcinoma cases.

b Not all cases had a bone scan prior to or shortly after the time of surgery.

c PSA data were unavailable for 36 cases at the time of diagnosis. PSA value is in nanograms per millilitre (ng ml^−1^).
